# Production of high-affinity glycosylated anti-mouse conjugated nanobodies in *Pichia pastoris*

**DOI:** 10.3389/fbioe.2025.1673481

**Published:** 2025-10-15

**Authors:** Sofía Orioli, Javier Santos, Lorena I. Ibañez, Cecilia D’Alessio

**Affiliations:** ^1^ Universidad de Buenos Aires, Facultad de Ciencias Exactas y Naturales, Departamento de Fisiología y Biología Molecular y Celular, Instituto de Biociencias, Biotecnología y Biología Traslacional (iB3), Buenos Aires, Argentina; ^2^ Universidad de Buenos Aires, Facultad de Ciencias Exactas y Naturales, Departamento de Química Biológica, Buenos Aires, Argentina; ^3^ Consejo Nacional de Investigaciones Científicas y Técnicas (CONICET), Buenos Aires, Argentina; ^4^ CONICET- Universidad de Buenos Aires. Instituto de Química Física de los Materiales, Medio Ambiente y Energía (INQUIMAE), Buenos Aires, Argentina

**Keywords:** nanobody, *Pichia pastoris*, glycosylation, mouse kappa light chain, recombinant expression

## Abstract

**Introduction:**

Nanobodies (NBs) are small antibody fragments derived from camelid heavy-chain antibodies, which represent the minimal functional domain capable of antigen recognition and binding. NBs are 10 times smaller than conventional antibodies, exhibit a compact structure, and have high stability, making them ideal for recombinant production. The eukaryotic unicellular system *Pichia pastoris* provides multiple advantages for protein expression, including the ability to perform several eukaryotic post-translational modifications such as glycosylation.

**Methods:**

In this work, we engineered a modular plasmid sequence that, through specific restriction enzyme cuts and ligations, codes the expression of a secreted anti-mouse *kappa* chain NB fused with various accessory peptides in *P. pastoris*. This system enables the incorporation of a plastic binding sequence for immobilization onto polystyrene surfaces, a histidine tag (Hisx6) for purification, the horseradish peroxidase (HRP) enzyme for chemiluminescence detection, or the biotinylatable AviTag sequence for detection using a different method, in multiple combinations.

**Results:**

We successfully expressed and purified anti-*kappa* NBs fused to a Hisx6-tag (κNB) and HRP–Hisx6-tag (κNB–HRP), with subsequent structural and functional characterization revealing high affinity and specificity for mouse immunoglobulins. The κNB–*kappa* light chain domain complex was modeled, showing a fitted surface interaction of the CDR3 domain. The position of a glycan present in κNB CDR3 within the complex was modeled, predicting that glycan addition would not affect the interaction surface. Accordingly, no functional differences were observed in κNB after deglycosylation, indicating that high mannose glycan addition has not interfered with its binding capability. Glycosylated and deglycosylated κNBs fused to HRP were produced with retained HRP activity and proved to be functional as secondary antibodies.

**Discussion:**

Our results show the *P. pastoris* eukaryotic system’s versatility in producing NBs and conjugated NBs with or without post-translational modifications that may be required for diverse biotechnological applications.

## Introduction

**FIGURE 1:**
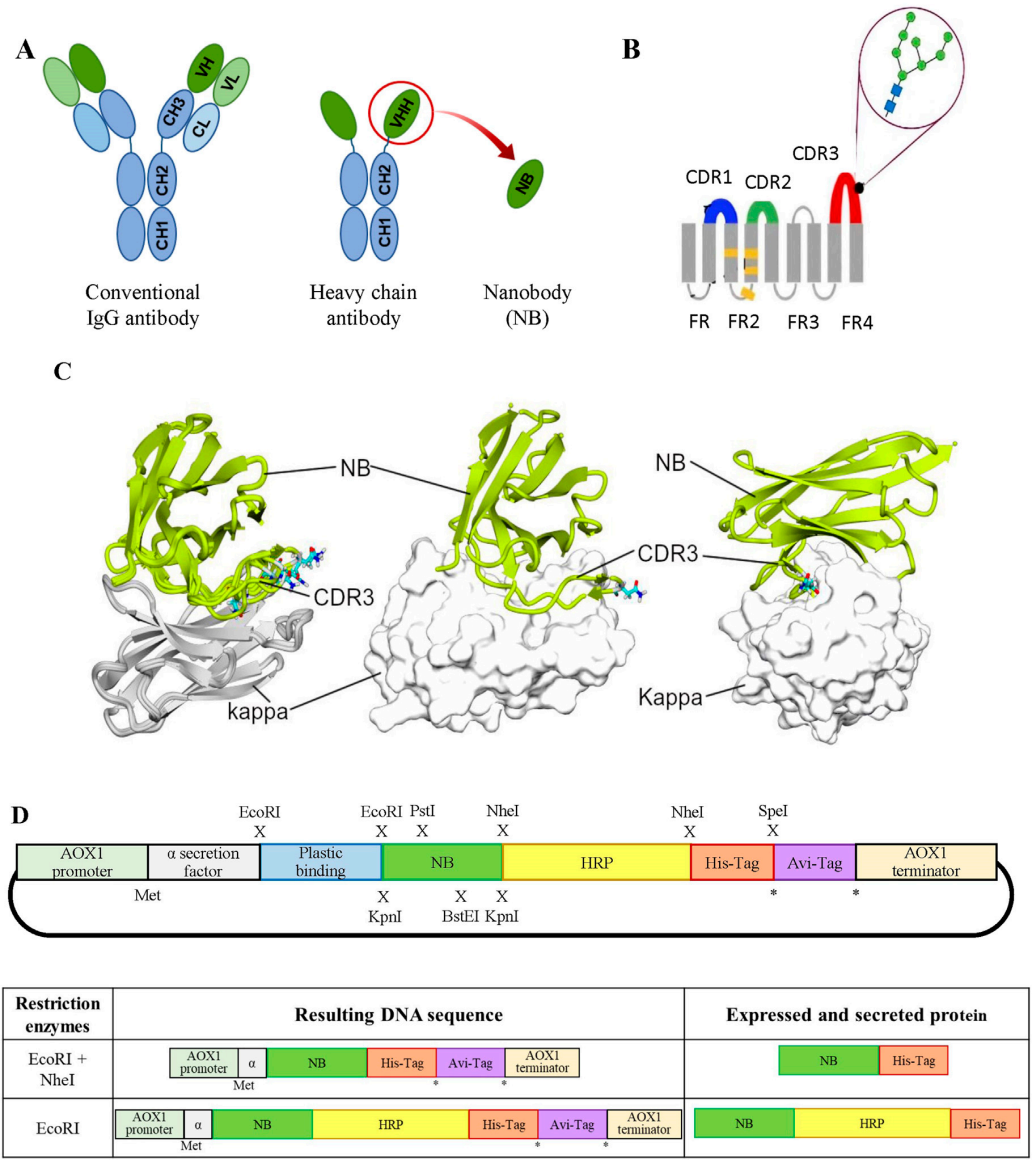
**(A)** Schematic representation of different antibody formats. On the left, a typical mammalian IgG antibody; in the center, a camelid heavy-chain IgG; and on the right, a VHH or NB. The acronyms stand as follows: CH, heavy-chain constant domain; CL, light-chain constant domain; VH, heavy-chain variable domain; and VL, light-chain variable domain. **(B)** NB diagram showing the positions of CDR domains: CDR1 (blue), CDR2 (green), and CDR3 (red) and the predicted position of a single *N*-glycan at N107 (figure adapted from Mitchell and Colwell (2018)). **(C)** Interaction of κNB with the mouse *kappa* light chain. In the left panel, a superimposition of five AlphaFold3 models is shown interacting with the mouse *kappa* light chain (gray, *kappa*; green, κNB). The predicted glycosylated Asn107 in each model are depicted in sticks. In the central panel, only one model is presented, with the *kappa* chain shown in surface representation, similar to the right panel, where the model’s orientation was changed to highlight the extensive interaction of CDR3. **(D)** *Upper panel:* schematic representation of the modular pPICZαA vector designed for the expression of an NB fused to different accessory peptides/proteins. From 5′ to 3’: AOX1 promoter to drive expression, *Saccharomyces cerevisiae* mating factor α secretion signal, plastic-binding sequence, exchangeable NB, HRP, Hisx6 tag, AviTag sequence, and at the AOX1 transcription terminator. Restriction enzyme sites introduced at the indicated positions (x) allow the generation of different NB variants. The asterisk (*) indicates alternative translation stop codons, while (Met) marks the translation initiation site. *Lower panel*: on the left, DNAs obtained after restriction digestion and re-ligation of the modular vector; on the right, the corresponding mature protein sequences after translation and removal of the α-factor signal.

Nanobodies (NBs) are the smallest antibody fragments capable of recognizing and binding antigens with high specificity and affinity (De Marco, 2020; Kolkman and Law, 2010). NBs consist solely of the ∼15 kDa heavy-chain variable region (V_HH_) of homodimeric heavy-chain antibodies discovered in camelids in the 1990s (Muyldermans, 2013; Hamers-Casterman et al., 1993) (Figure 1A). Due to their small size, NBs possess high chemical, thermal, and structural stability; high solubility; and the ability to bind to epitopes that are difficult to access for larger conventional antibodies (Kolkman and Law, 2010; Muyldermans, 2021). The aforementioned properties, along with their simple structure, make NBs excellent candidates for recombinant expression in microorganisms.

*Escherichia coli* is the most commonly used host for NB production in laboratories. However, both cytoplasmic expression and periplasmic secretion present significant challenges. In the periplasm, the limited compartment size can become overcrowded when NBs are highly expressed, overwhelming the chaperone system and impairing proper protein folding. In the cytoplasm, the reducing environment hinders disulfide bond formation, often resulting in NB aggregation into inclusion bodies, which cannot always be successfully refolded (De Marco, 2020; Liu and Huang, 2018). Moreover, generating a conjugated NB whose partner, such as horseradish peroxidase (HRP), is not efficiently expressed in bacterial systems would present a bottleneck (Spadiut and Herwig, 2013; Capone et al., 2015). The methylotrophic yeast *Pichia pastoris* has emerged as a popular eukaryotic expression system as it can perform post-translational modifications such as *N*- and *O*-glycosylation, disulfide-bond formation, and protein folding in the secretory pathway (Cregg et al., 2000), while remaining easy to genetically modify and culture. Its highly efficient secretory pathway facilitates protein purification from culture supernatants when expressed proteins are fused to secretion peptides. Although several promoters have been used in expression vectors, the AOX1 (alcohol oxidase 1) promoter—inducible by the addition of methanol—is still very popular as it enables high, stable, and controlled expression of recombinant proteins (Cregg et al., 2000; Bernauer et al., 2021; Sun et al., 2019).

Given that ∼99% of commercial mouse antibodies contain the *kappa* light chain (Pleiner et al., 2018), an anti-*kappa* NB (κNB) is highly valuable for various applications such as immunoassays, Western blots, and ELISA. Generating in-house conjugated variants of anti-mouse *kappa* chain NBs from a single DNA sequence will enable diverse applications in research and diagnostics.

In this work, we expressed functional anti-mouse *kappa* chain NBs [referenced as TP1170 in Pleiner et al. (2018)] in the methylotrophic yeast *P. pastoris* using a single modular DNA sequence that, through differential cuts and ligations, allows obtaining the NBs fused to diverse accessory peptides that ease detection and purification. We showed that the expression of glycosylated κNB in fusion with HRP was successful and that glycosylation did not affect the binding capability of κNB or κNB–HRP to the mouse kappa light chain. Glycans may be removed for homogeneity purposes after purification without loss of functionality.

## Results

### Interaction of κNB with the mouse immunoglobulin *kappa* light chain

AlphaFold3 was used to predict the structure of the complex between the κNB and the mouse *kappa* variable domain. It is worth mentioning that the κNB sequence contains one consensus sequence for *N*-glycosylation (N-X-S/T, where X can be any amino acid except P), which occurs at N107 in CDR3 of κNB (Figure 1B). The predicted model for the κNB–*kappa* complex yielded interface predicted template modeling score (ipTM) and predicted template modeling score (pTM) values of 0.71 and 0.78, respectively, indicating that the structure could be similar to the actual structure. The most inaccurate region of the model was the CDR3 stretch of the κNB, which, paradoxically, was involved in the direct interaction between κNB and the *kappa* domain. Interestingly, the NXS/T glycosylation site located in the CDR3 region was exposed to the solvent in four of five models, suggesting that if CDR3 adopted these conformations, the glycan would not disrupt the interaction between the κNB and *kappa* domains. It is worth noting that the CDR3 region creates a large contact area between both domains, which could contribute to the complex’s high stability (Figure 1C).

The nature of the interface between subunits was examined using the PISA tool [Protein Interfaces, Surfaces, and Assemblies, https://www.ebi.ac.uk/msd-srv/prot_int/cgi-bin/piserver Krissinel and Henrick (2007)]. The interface between the κNB (A) and its target mouse *kappa* variable domain (B) involved 26 and 27 residues, respectively, resulting in an interface area of 875.2 Å^2^ (13.1% and 14.5% of the total surface areas of the proteins, respectively). The ΔG value (a measure of the solvation-free energy) of −10.4 kcal/mol (P-value = 0.169) indicates stabilization upon complex formation with a highly hydrophobic interface, as expected for a specific interaction, suggesting affinity. Three inter-chain hydrogen bonds can be found at the interface (A: Tyr113 and B: Glu58; A: Arg45 and B: Gln62; and A: Thr116 and B: Ser67), which are also predicted to contribute to complex stabilization.

Taken together, these results indicate that κNB may form a stable and specific complex with the mouse *kappa* variable domain, with the CDR3 region contributing substantially to the interface and the NXS/T glycosylation site remaining solvent-exposed and non-disruptive to binding.

### Modular plasmid design for the expression of κNBs fused to different accessory peptides

Using pPICZαA as the vector backbone, a unique vector was designed for the expression in *P. pastoris* of an anti-κNB fused to different tags and accessory peptides, providing a variety of features, such as a plastic-binding sequence, HRP, the biotinylatable AviTag sequence, and a 6-histidine tag under the control of the methanol-inducible AOX promoter (Supplementary Figure S1). Each coding sequence was flanked by the sequences of different restriction enzymes to allow the specific removal of each individual segment, after which the plasmid can be re-ligated and different nanobody variants expressed (Figure 1D). All sequences were preceded by a *Saccharomyces cerevisiae* mating factor α secretion signal, used to secrete the expressed protein to the yeast culture supernatant. The final designed modular vector contained the following coding sequences: a plastic binding peptide (flanked by EcoRI sites); an anti-mouse κNB (flanked by KpnI, PstI, and BstEI sites); a HRP (flanked by NheI sites); a Hisx6 tag (after which there is a stop codon followed by a SpeI site); and finally, a biotinylatable AviTag sequence, also followed by a stop codon. As restriction with NheI and SpeI generates compatible cohesive ends, using both enzymes allows for the removal of HRP and the Hisx6 tag, resulting in the expression of the NB fused to the AviTag sequence. Similarly, restriction with EcoRI followed by re-ligation would remove the possibility of binding the protein to plastic, leaving only the Hisx6 tag to facilitate purification. The new modular vector was named pPICZαA-Plastic-κNB-HRP-Hisx6-AviTag (henceforth referred to as “modular vector”).

Restriction reactions, re-ligations, and amplifications were carried out to obtain different variations of the plasmid. The HRP detection sequence was removed with NheI, and the plastic binding sequence with EcoRI, resulting in a plasmid for the expression of κNB fused only to the Hisx6 tag to test the system (κNB). Simultaneously, a vector for the expression of the κNB fused to HRP and the Hisx6 tag was obtained by restriction only with EcoRI (κNB–HRP). All new plasmids were verified by sequencing and integrated into the *P. pastoris* X-33 genome.

### Expression and characterization of κNB expressed in *Pichia pastoris*

**FIGURE 2:**
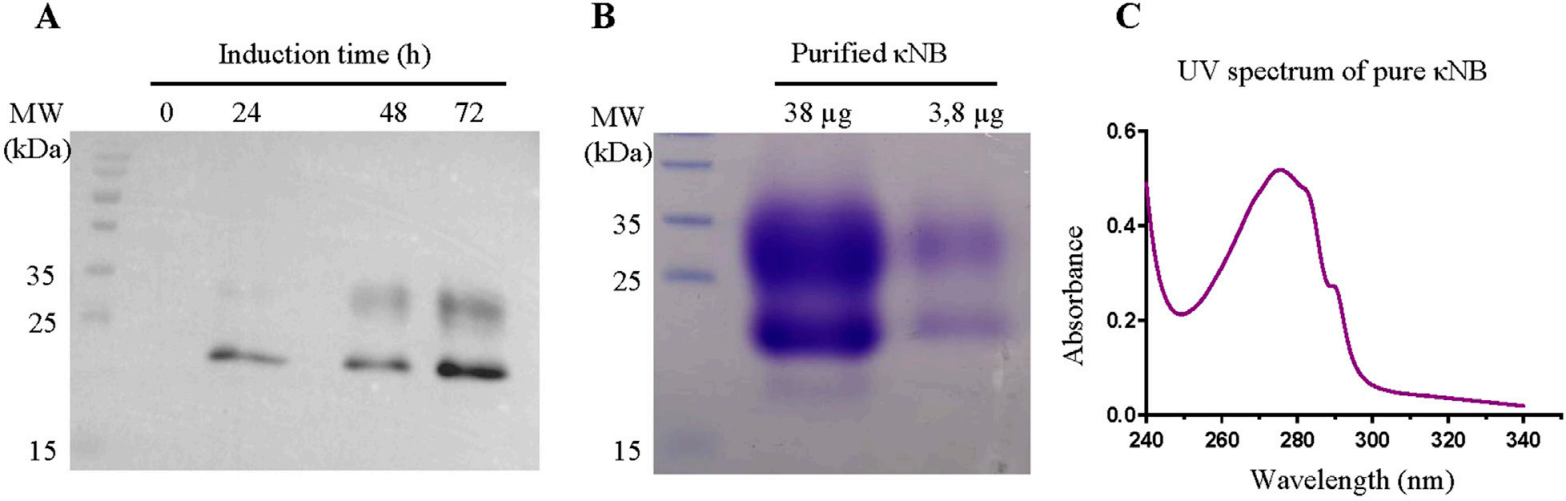
**(A)** κNB expression in *Pichia pastoris*-induced supernatants. TCA-precipitated supernatants (300 µL) were run on a 15% SDS-PAGE, transferred to a PVDF membrane, and incubated with a mouse anti-His primary antibody (1:7500) and with an anti-mouse conjugated to HRP (1:15,000) secondary antibody. The image obtained is the result of the superposition of the Western blot with the image taken from the pre-stained marker (MW). Lanes to the right correspond to 0, 24, 48, and 72 h of culture induction. **(B)** SDS-PAGE (15%) of pure κNB (loaded 38 μg and 3.8 µg of protein). The expected κNB size is 15.9 kDa. **(C)** UV–visible spectrum of a 3.8 µg of κNB after subtracting the buffer alone spectrum.

Expression of κNB was carried out in a 200 mL culture of the *P. pastoris* strain X-33 transformed with the plasmid digested with NheI and EcoRI and re-ligated. Inductions were performed with 1% methanol and every 24 h, aliquots were taken, and proteins were precipitated with 15% trichloroacetic acid. The presence of κNB was confirmed by Western blot using a mouse anti-His primary antibody, followed by an anti-mouse-HRP secondary antibody (Figure 2A). A progressive increase in protein quantity over time was observed with a maximum of 72 h of methanol induction. κNB has an expected molecular mass of 15.9 kDa. However, two larger, diffuse bands between 20 and 30 kDa were observed.

Protein produced in 200 mL of the supernatant of cultures induced during 72 h was purified by affinity chromatography using a 1-mL Ni-NTA column. Bound κNB was eluted with 300 mM imidazole, and fractions in which eluted NBs were identified were pooled and dialyzed against TBS. Sodium dodecyl sulfate polyacrylamide gel electrophoresis (SDS-PAGE) and staining with Coomassie Brilliant blue after κNB purification and dialysis revealed the same two-band pattern previously shown through Western blot (Figure 2B).

The UV spectrum of the pure protein corresponds to the expected profile, considering the number of tryptophan and tyrosine residues in the sequence (Figure 2C). Protein concentration was quantified using the absorbance at 280 nm and the extinction coefficient of 32,890 M^-1^ cm^-1^, resulting in a pool of 2.37 mg/mL and, by extrapolation, a yield of 17.8 mg/L of culture.

**FIGURE 3:**
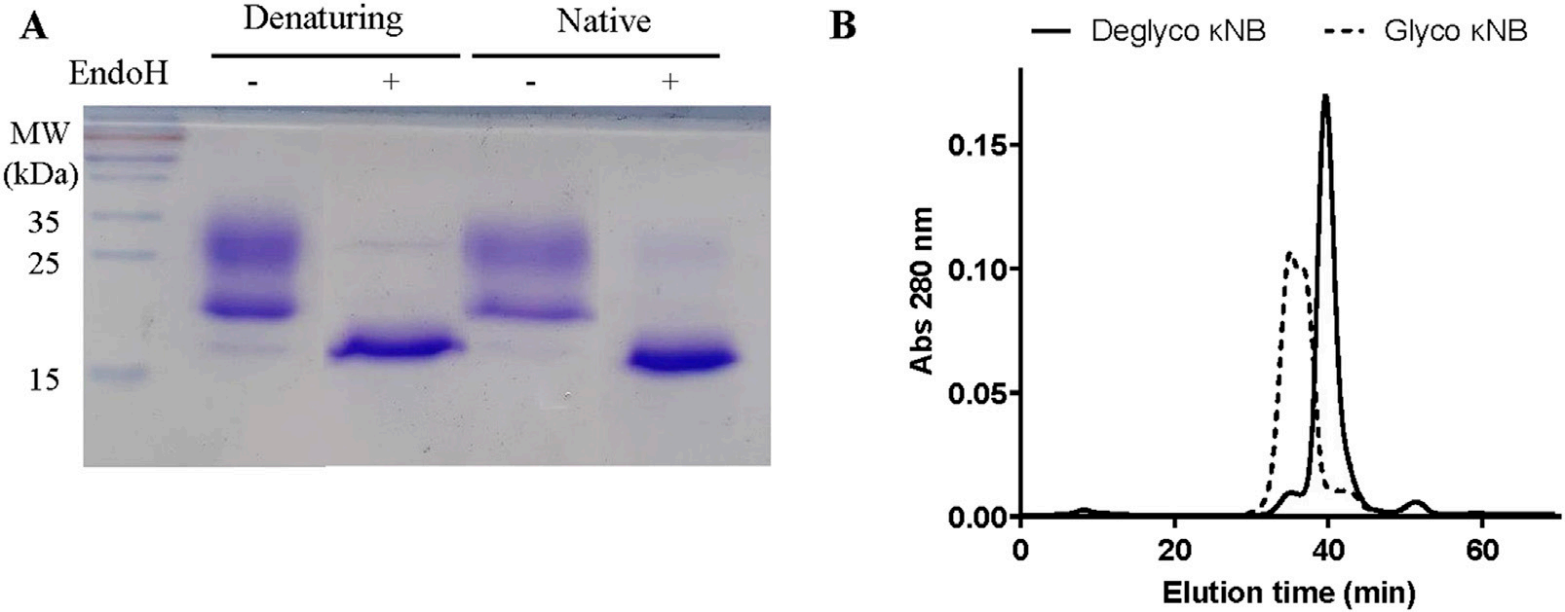
**(A)** Analysis of the glycosylation status of κNB expressed in *Pichia pastoris*. κNB was deglycosylated with EndoH under both denaturing and native conditions and analyzed on 15% SDS-PAGE. **(B)** SEC-HPLC profile of glycosylated and deglycosylated κNBs under native conditions. The graph represents the elution chromatogram of the different molecules, plotted as absorbance at 280 nm vs. elution time. Each sample was run for 70 min.

The presence of the two diffuse bands of a lower mobility than the predicted molecular weight observed in Figures 2A,B was attributed to the possible glycosylation of Asn 107 located in CDR3 of the κNB during its transit through the yeast secretory pathway. Two κNB deglycosylation reactions were carried out to confirm this: one under native conditions and another under denaturing conditions, using endoglycosidase H (EndoH) to cleave high mannose glycans produced by yeast. The reactions were analyzed using a new SDS-PAGE, along with untreated controls (Figure 3A), showing that after treatment with EndoH, the two diffuse bands become one concise and defined band of the expected size of 15.9 kDa. Based on these results, it was concluded that the presence of the two-band pattern was due to the *N*-glycosylation of Asn 107 of κNB. Although AlphaFold3’s prediction of the κNB-kappa light chain complex structure suggested that the presence of an *N*-glycan would not affect NB-*kappa* domain interaction, we evaluated how κNB’s performance and/or function would be affected by the presence of glycans. It is worth mentioning that a third, very faint band, with the same apparent mobility of 15.9 kDa as deglycosylated κNB, was visible in the controls. This indicates that a small fraction of the original κNB may have remained non-glycosylated and that *P*. *pastoris* can produce different glycosylation patterns in the same protein.

SEC-HPLC was carried out on both glycosylated and deglycosylated κNBs under native conditions to evaluate purity, size, and aggregation. The elution profile of deglycosylated κNB (Figure 3B) displayed a well-defined, narrow elution curve, with a peak at 40 min. On the other hand, the SEC profile of glycosylated κNB showed an earlier elution peak at 35 min, consistent with the presence of a larger molecule. Moreover, a wider peak was obtained for glycosylated κNB, likely due to the heterogeneous glycosylation profile revealed both through SDS-PAGE and Western blot.

### κNB functional tests

**FIGURE 4:**
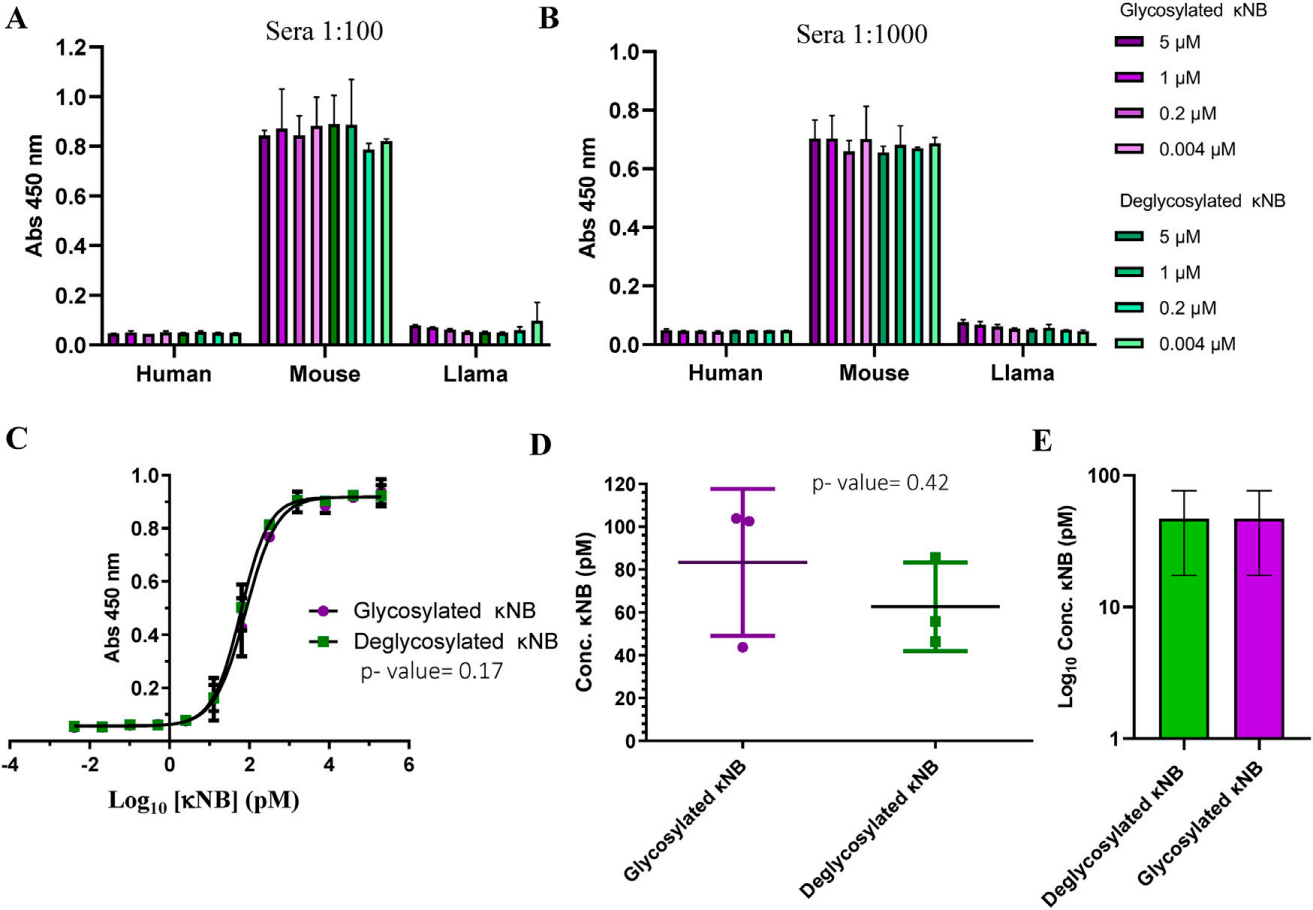
κNB specificity toward mouse antibodies. Graph depicting κNB binding profile to an ELISA plate coated with human, mouse, and llama sera in **(A)** 1:100 dilution and **(B)** 1:1000 dilution. Absorbance of the TMB substrate was measured at 450 nm. **(C)** κNB detection limit of 1:6000 dilution of mouse serum. Absorbance was measured at 450 nm. Log_10_ of κNB concentrations was used to improve visualization. **(D)** Comparison of EC_50_ of glycosylated and deglycosylated κNBs. Standard deviations and p-value determined after a t-test are indicated. **(E)** Comparison of limits of detection determined for both glycosylated and deglycosylated κNBs. The limit of detection was defined as the highest concentration of κNB that produces a signal at 450 nm that is greater than the cut-off value set at ×3 times the blank. Log_10_ of κNB concentrations was used to improve visualization.

The functionality and specificity of the anti-*kappa* NB to recognize mouse antibodies were assessed through ELISA. A plate was coated in duplicate with human, llama, and mouse sera at 1:100 (Figure 4A) and 1:1000 (Figure 4B) dilutions, followed by incubation with serial dilutions of both glycosylated and deglycosylated κNBs to assess whether the presence of glycans in the κNB affects antibody recognition. The binding of κNB to the kappa light chain in the sera was detected using an anti-His-HRP antibody. Resulting data from absorbance at 450 nm showed that the κNB specifically recognized only mouse serum, without showing any detectable cross-reactions with sera from human or llama origins, even at higher IgG concentrations (as in 1:100 dilution), indicating that the κNB is highly species-specific. No differences were observed between glycosylated and deglycosylated κNBs in IgG recognition up to a concentation of 0.004 μM.

To determine κNB detection limits, an ELISA was carried out using a 1:6,000 dilution of mouse serum for coating and incubating with 200 nM to 4 fM serial dilutions of glycosylated and deglycosylated κNBs. In this case, a higher mouse serum dilution was used because the results from Figures 4A,B suggested that the surface of the wells might be saturated at 1:100 and 1:1000 dilutions. The interaction curve obtained after absorbance measurement showed that both forms of the κNB displayed identical performances (Figure 4C). To determine whether the differences between κNBs were statistically significant, an analysis of variance (ANOVA) was carried out, defining a significance level (α) of 5%. The resulting *p*-value of 0.17 confirmed that there are no significant differences between the glycosylated and deglycosylated κNBs, and therefore, κNB performance is not affected by the presence or absence of glycans, provided that they are removed *after* protein synthesis.

The κNB’s EC_50_ (effective concentration, which was defined as the concentration at which 50% of the antibody is bound) was determined to be 80.9 pM for the glycosylated κNB and 61.2 pM for the deglycosylated κNB (difference determined not significant using a t-test, *p*-value 0.42) (Figure 4D).

The limits of detection were determined for both glycosylated and deglycosylated forms of the κNB. These were defined as the highest κNB concentration that gives a signal at 450 nm higher than the cut-off value, set at ×3 times the blank of the reaction. Using this criterion, the limit of detection was 46.9 pM for both forms of κNB (Figure 4E).

Altogether, our results show that glycosylation did not affect κNB functionality or specificity as glycosylated and deglycosylated κNBs behaved indistinguishable one from another.

### κNB–HRP expression in *P. pastoris* and functional tests

After confirming that the smallest and simplest variant of κNB expressed in *Pichia pastoris* was specific, functional, and sensitive, the yeast was subsequently transformed with a plasmid encoding a κNB construct fused to horseradish peroxidase (HRP) and a His_6_ tag (κNB–HRP). Integration of the gene in the genome was confirmed by PCR, and induction of several clones was carried out in 10 mL cultures to quickly assess HRP enzyme activity in culture supernatants. The best clone that displayed the highest HRP activity as measured using a fast activity test (see *Methods*) was chosen for the induction of a 200 mL *P. pastoris* culture. κNB–HRP was purified as described for κNB using a Ni-NTA affinity column. Quantification by absorbance of the eluted and dialyzed pool was measured at 280 nm using an extinction coefficient of 45,840 M^-1^ cm^-1^. A concentration of 759.8 µg/mL was obtained for the purified pool, resulting in a total yield of 7.2 mg κNB–HRP per liter of total culture.

**FIGURE 5:**
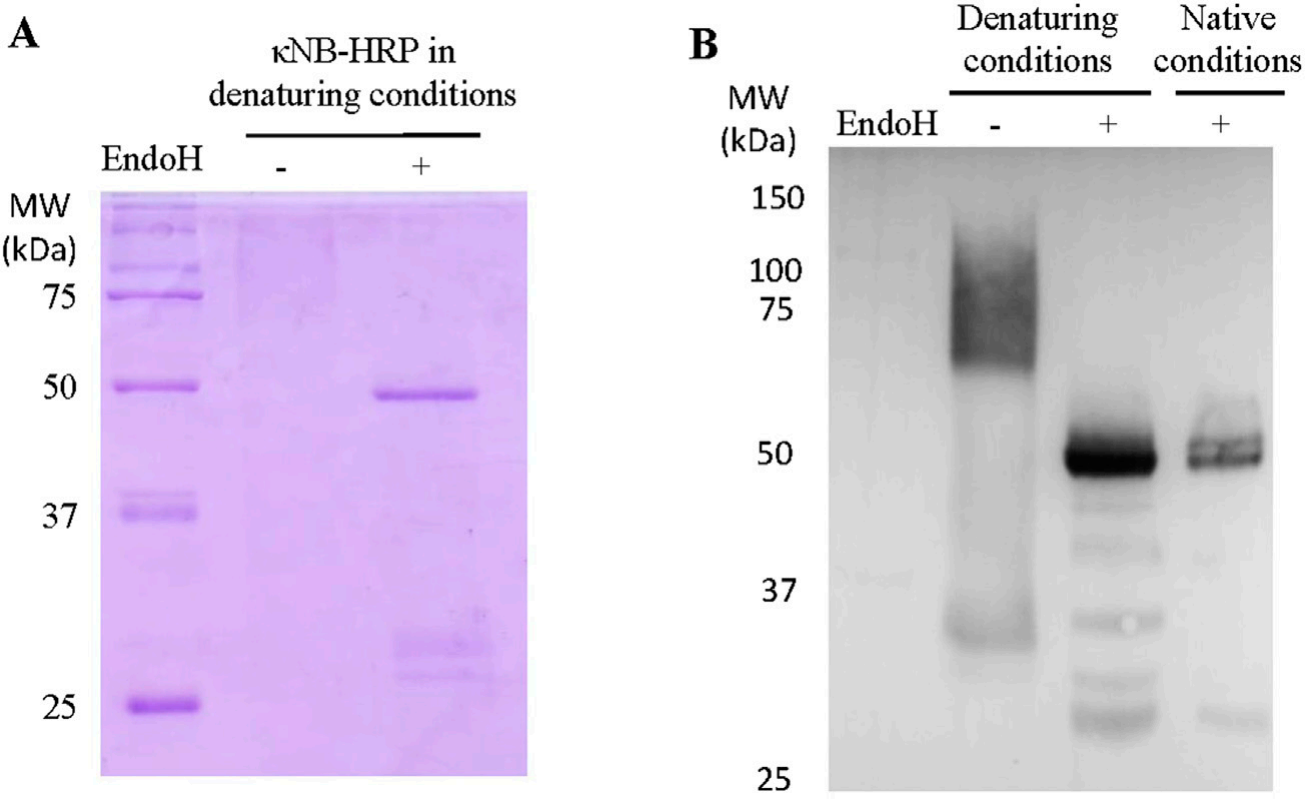
Expression of κNB-HRP in *Pichia pastoris*. **(A)** SDS-PAGE 10% of pure κNB–HRP (5 μg) before and after treatment with EndoH under denaturing conditions. **(B)** Western blot performed on κNB-HRP (5 μg) after deglycosylation under both native and denaturing conditions. Untreated controls were included. Mouse anti-His was used as the primary antibody (1:7500) and anti-mouse-HRP as the secondary antibody (1:15,000).

Similarly to the expression of κNB, κNB–HRP was expressed with a highly diffuse and heterogeneous pattern ranging from 60 to 100 kDa using SDS-PAGE (Figure 5A). These diffuse bands were more visible in a Western blot using the anti-His antibody (Figure 5B), which confirmed κNB identity. The diffuse bands were sharpened to a single band of the expected size of 49 kDa after deglycosylation under both denaturing (Figures 5A,B) and native conditions (Figure 5B) with EndoH. A few smaller bands than expected were observed both through SDS-PAGE and Western blot upon deglycosylation and are probably due to some extent of protein degradation.

**FIGURE 6:**
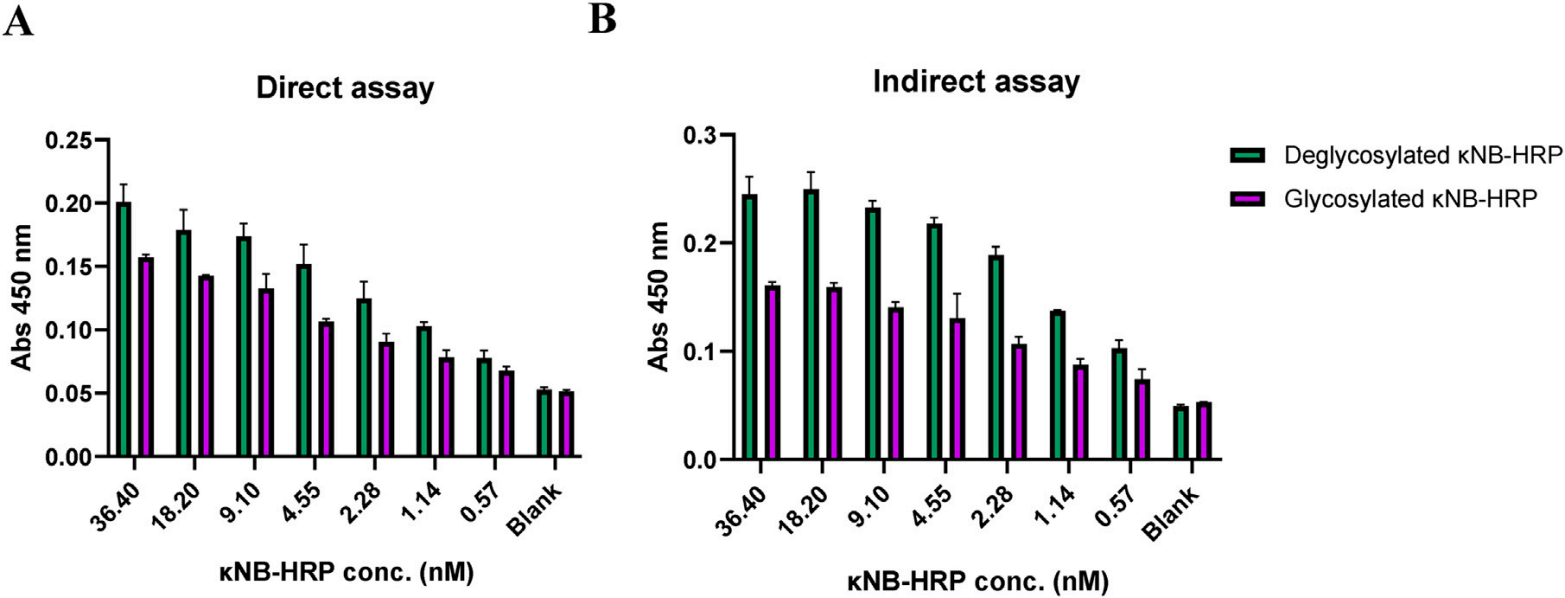
Interaction profile by ELISA of κNB–HRP with **(A)** mouse serum in a direct assay and **(B)** RBD protein and anti-RBD mouse serum in an indirect assay. κNB–HRP was tested in concentrations ranging from 36.4 to 0.5 nM, and absorbance was measured at 450 nm.

An ELISA was carried out to test the κNB–HRP functionality. The plate was coated with both mouse sera, for a direct assay with κNB–HRP (Figure 6A), and with SARS-CoV2 receptor-binding domain (RBD) protein for an indirect assay using mouse anti-RBD serum (Arbe et al., 2020; Idrovo-Hidalgo et al., 2024) as the primary antibody and κNB-HRP as the secondary antibody (Figure 6B). κNB–HRP and κNB–HRP deglycosylated under native conditions were tested in the range of 36–0.5 nM. In both assays, both glycosylated and deglycosylated κNB–HRPs produced measurable signals at 450 nm, with the deglycosylated molecule producing higher average signal values. ANOVA produced a *p*-value lower than that of the α-set at 5% for both tested conditions (*p* = 1.87 × 10^7^ for the assay against mouse serum and 1.93 × 10^11^ against RBD and mouse anti-RBD serum); therefore, in this case, the difference in performance between the glycosylated and deglycosylated κNB–HRPs is statistically significant, indicating that the presence of glycans in both κNB and HRP domains did not affect the function and, in some cases, improved the performance.

**FIGURE 7:**
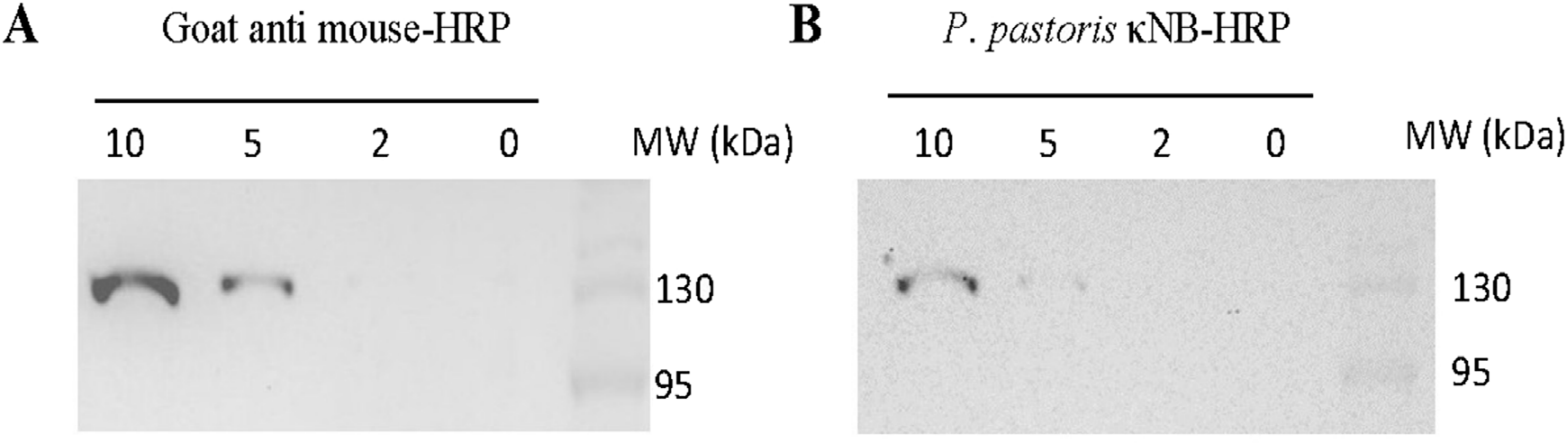
Use of κNB–HRP as a secondary antibody in a Western blot. Samples from 10 through 0 µg of total protein extracts expressing a control GFP fusion protein were tested. Membranes were incubated with mouse anti-GFP (1:1,000) as the primary antibody and **(A)** goat anti-mouse-HRP of known performance (1:15,000) or **(B)** deglycosylated, under native conditions, κNB-HRP (1:3000) as the secondary antibody.

κNB–HRP’s performance as a secondary antibody was also tested in a dot blot (Supplementary Figure S2) and a Western blot (Figure 7). The dot blot demonstrated the ability of κNB–HRP to function as a secondary antibody when pure RBD was spotted onto a membrane, followed by incubation with anti-RBD mouse serum as the primary antibody. No signal was observed in the absence of the primary serum, indicating that no nonspecific signals are generated using κNB–HRP. In a Western blot, different amounts of a total protein extract of the yeast *Schizosaccharomyces pombe* expressing a GFP–glucosidase I fusion protein were run in an SDS-PAGE, and the blocked membrane was incubated with a mouse anti-GFP primary antibody (1:1000). The membrane was divided: one half was used as a methodological control and incubated with a commercial anti mouse-HRP secondary antibody diluted 1:15,000 (Figure 7A); the second half was incubated with the deglycosylated (native conditions) κNB–HRP diluted 1:3,000 (Figure 7B). Both membranes showed the expected band of the fusion control protein at approximately 120 kDa and were able to detect 5–10 µg of total protein with the chosen dilutions. Although the κNB–HRP concentration used (0.15 ng/μL) cannot be directly compared with the commercial antibody (of unknown concentration), both were tested at working dilutions in which they are functional, allowing us to demonstrate that κNB–HRP can effectively be used as a secondary antibody in ELISA (Figure 6), dot blot (Supplementary Figure S2), and Western blot assays (Figure 7).

## Discussion

The discovery of NBs 30 years ago revolutionized antibody engineering due to their structural characteristics and the advantages they conferred over conventional antibodies. These benefits have proven useful for the development of potential applications in therapeutic, diagnostic, and research areas. Although expression of NB is popular in bacterial systems, expression in eukaryotic systems would allow post-translational modifications of both NBs and NB-fusion proteins or tags that may require glycans to fold properly.

In this work, a modular plasmid was designed for the expression in *P. pastoris* of an anti-mouse *kappa* chain NB fused to various accessory sequences of choice, which could be used in immunoassays as a secondary antibody, for immunoprecipitations, or for antigen capture in ELISA tests, with tags to facilitate NB purification and/or detection.

The AlphaFold prediction shows that the κNB–*kappa* light chain complex is compact and well-structured, with a large CDR3 stretch of κNB being involved in the direct interaction. Moreover, we predicted that the presence of a glycan in the CDR3 would not affect the interaction surface. Further work will be carried out to investigate the internal motions of the CDR3 stretch to evaluate whether binding depends on the local dynamics of this region.

The modular vector was cloned into *E. coli*, and three derived vectors were obtained: one carrying the whole modular plasmid (pPICZαA-Plastic-κNB-HRP-Hisx6-Avitag), one for the expression of κNB without any accessory tags, and one for the expression of κNB–HRP. The κNB and κNB–HRP plasmids were integrated into the genome of *P. pastoris*, and NBs were expressed as secreted proteins under the AOX1 methanol-induced promoter.

Anti-*kappa* mouse NB was purified in its monomeric form from the culture supernatant using an Ni-NTA affinity column, with a yield of 17.8 mg/L, one of the highest values reported for the expression of NBs in an expression system that has the advantage of eukaryotic post-translational modifications (Baghban et al., 2016; Ezzine et al., 2012). The κNB was glycosylated, a fact confirmed through treatment of pure protein with endoglycosidase H. The glycosylated and non-glycosylated preparations were analyzed through Western blot and SEC-HPLC, confirming that the size heterogeneity was due to the glycosylation in the eukaryotic system.

The specificity and functionality of glycosylated and non-glycosylated κNBs were tested using ELISA. These assays demonstrated the specificity of both glycoforms of κNBs, which interacted only with mouse serum and not with human or llama sera. The detection limits, EC_50_ values, and variance analysis revealed no statistically significant differences in performance between the glycosylated and non-glycosylated NBs (with a p-value of 0.17), indicating that, as predicted, the presence of *N*-glycans did not affect their functionality.

*N*-glycosylation is involved in the quality control of glycoprotein folding in the endoplasmic reticulum (D’Alessio et al., 2010). The presence of a glycan in the molecule in that compartment favors that only properly folded glycoproteins follow the secretory pathway of eukaryotic cells and thus are secreted to the culture media; otherwise, they are retrotranslocated to the cytosol from the endoplasmic reticulum and degraded. This ensures the native folding status of proteins obtained from the culture’s supernatants. Deglycosylation resulted in a more homogeneous profile, indicating that expression in a system that facilitates glycosylation (such as *P. pastoris*) can be beneficial for obtaining higher yields of correctly folded proteins and that any resulting heterogeneity can subsequently be removed if needed for regulatory purposes.

The second NB variant induced in *P. pastoris* and purified from the modular construction was κNB–HRP to produce a useful secondary antibody replacement. The protein was obtained at a calculated yield of 7.2 mg of protein/L of culture. κNB–HRP proved to be functional after successful trials in both ELISA and Western blot. κNB and HRP both have glycosylation sites, so expression in *P. pastoris* would result in both “domains” being glycosylated. However, it was shown that the deglycosylated version of κNB–HRP had a slightly better performance than its glycosylated counterpart. It is worth mentioning that although HRP is expressed in prokaryotic systems, such as *E. coli*, several difficulties are reported: affected stability due to the lack of glycosylation, low refolding efficiency from inclusion bodies, and reduced catalytic activity after refolding (Gundinger and Spadiut, 2017), all of which position *P. pastoris* as a great tool to express different conjugated NBs, such as NB–HRP fusions.

Previous work from our group has shown that there were no structural differences between glycosylated and deglycosylated Spike’s RBDs and that deglycosylated RBDs elicited stronger humoral and cellular responses when used as an immunogen in mice (Idrovo-Hidalgo et al., 2024). Moreover, we demonstrated that deglycosylated RBD performed better than human-expressed RBD as an antigen in ELISA to distinguish negative from positive human sera. Other studies have reported that the absence of glycosylation can reduce antibody recognition, although no structural analyses have been performed to confirm proper folding after glycan removal (Schwarze et al., 2024). In the present work, both glycosylated and deglycosylated κNBs and κNB–HRPs were tested in binding assays, with the deglycosylated κNB-HRP yielding slightly higher absorbance signals. Taken together, these observations emphasize the importance of evaluating both glycosylated and deglycosylated versions of recombinant proteins as functional differences may arise depending on the context and application.

As proof of concept, the modular vector was used to produce κNBs either alone or fused to HRP, both of which were functional and able to bind the mouse *kappa* light chain. This modular vector for *P. pastoris* expression is a useful tool with the potential for broader applications in the future—such as improving the orientation and immobilization of NBs on the polystyrene surfaces of ELISA plates or enabling biotinylation-based modifications that could expand the range of κNB applications. Moreover, it is important to mention that the high cost and significant delays associated with importing research antibodies hinder access to these crucial reagents in many low-income countries. Establishing a local, efficient NB production system could offer a valuable solution to reduce costs and wait times, fostering local research and development.

## Experimental procedures

### Materials

The yeast extract, tryptone, and agar were purchased from Britania (Argentina). Yeast nitrogen base and peptone were obtained from Difco. Zeocin, Taq polymerase, and dNTPs were obtained from Invitrogen, Waltham, Massachusetts, United States. Sorbitol, amino acids for culture media, and RNAse A were purchased from Sigma, Burlington, Massachusetts, United States. Dextrose was purchased from Biopack, Zárate, Argentina. DNA restriction enzymes and bovine seroalbumin (BSA) were obtained from New England Biolabs, Ipswich, Massachusetts, United States. T4 DNA Ligase was purchased from Promega, Madison, Wisconsin, United States. Oligonucleotides were synthesized by GenScript, Piscataway, New Jersey, United States. *SuperSignal® West Pico Chemiluminescent Substrate* for Western Blot and *SnakeSkin™ Dialysis Tubing* 10.000 MWCO for protein dialysis were acquired from Thermo Fisher Scientific, Waltham, Massachusetts, United States.

### Strains and media

The *Escherichia coli* DH5α strain was used for cloning and amplification purposes. *E. coli* growth was carried out at 37 °C in low salt LB medium (Luria–Bertani broth; 1% tryptone, 0.5% yeast extract, and 0.5% NaCl; pH 7.5). For the selection of transformed bacteria, zeocin was added up to a concentration of 25 μg/ml. The *P. pastoris* wild type strain X-33 (Invitrogen) was used for protein expression. Yeasts were grown in yeast extract peptone dextrose adenine (YPDA) medium (1% yeast extract, 2% peptone, 2% glucose, and 75 mg/L adenine). Transformed yeast cells were selected using yeast extract peptone dextrose sorbitol (YPDS) medium with zeocin (YPDA supplemented with 1M sorbitol and 100 μg/mL zeocin). For protein expression, cells were first grown in buffered glycerol complex (BMGY) medium (1% yeast extract, 2% peptone, 100 mM potassium phosphate buffer pH 6.0, 1% glycerol, 1.34% yeast nitrogen base, and 4 × 10^−5^% biotin). Protein expression was induced in buffered methanol complex (BMMY) medium (with the same composition as BMGY medium but in which glycerol is replaced by 1% methanol). Yeast cultures were grown at 28 °C. In all cases, solid plates were prepared by adding 2% agar to the respective medium.

### Prediction of the κNB–mouse *kappa* light chain complex structure

The structure of the complex between the nanobody (κNB) and the mouse *kappa* variable domain was predicted using AlphaFold3 at https://alphafoldserver.com/(Abramson et al., 2024). The sequence corresponding to the *kappa* domain (BBA57852.1) was obtained from the protein sequence database at NCBI. We used the sequence of the immunoglobulin *kappa* light chain variable region from *Mus musculus*. On the other hand, the sequence corresponding to the *kappa* light chain-binding nanobody, designated as TP1170 by Pleiner et al. (2018), was identified. Five different models of the complex were obtained and analyzed using standard parameters. For the analysis, pTM and ipTM scores were considered. The pTM score and the ipTM score are both derived from the template modeling (TM) score that measures the accuracy of the entire structure (Xu and Zhang, 2010; Zhang and Skolnick, 2004). A pTM score above 0.5 suggests that the predicted complex’s overall shape is probably similar to the real structure. The ipTM metric measures how accurately the relative positions of the subunits within the complex are predicted. Scores over 0.8 indicate reliable, high-quality predictions, while those below 0.6 point to possible prediction failure. ipTM values between 0.6 and 0.8 fall into a gray area where predictions may or may not be correct.

### Plasmid design

The individual coding sequences for an NB that recognizes the *kappa*-chain of mouse immunoglobulins (TP1170), a plastic-binding sequence (PB-TUP), the HRP enzyme (vHRP variant), and the AviTag sequence were previously described by Pleiner et al. (2018), Qiang et al. (2017), Yamagata and Sanes (2018), and Athavankar and Peterson (2003), respectively. The sequences were codon-optimized for expression in *P. pastoris* and designed to be incorporated into the pPICZαA vector (Invitrogen) using the molecular biology tool Benchling (https://www.benchling.com/). The sequences were arranged in a particular order with different restriction sites, allowing the removal of each accessory sequence and subsequent religation to generate vectors capable of expressing various combinations of fusion proteins. The construction was then synthesized by GenScript and cloned completely into the pPICZαA vector, resulting in a new secretion vector named pPICZαA-Plastic-κNB-HRP-Hisx6-Avitag (referred to as “Modular plasmid” in this work). Once synthesized, the vector was transformed into the *E. coli* DH5α strain and extracted by minipreparation. *E. coli* DH5α strains containing three derived vectors were obtained: one carrying the whole modular plasmid, one carrying a plasmid for the expression of κNB (κNB expression fused to a Hisx6 tag; pPICZαA-κNB-Hisx6) obtained upon cut with EcoRI, followed by religation and subsequent cut with NheI, followed by religation, and one for the expression of κNB–HRP (κNB in fusion to the HRP enzyme and a Hisx6 tag; pPICZαA-κNB-HRP-Hisx6) obtained upon cut with EcoRI, followed by religation.

### DNA procedures

*E. coli DH5*α chemocompetent cells were prepared as detailed by Sambrook and Russel (2001). After recovery, cells were plated in LB low-salt agar with zeocin 25 μg/mL and incubated overnight at 37 °C. Miniprep DNA isolations from bacteria were carried out as detailed by Sambrook and Russel (2001). When DNA was purified from 100 mL cultures, the GenElute™ HP Plasmid Midiprep Kit (Sigma-Aldrich) was used following the manufacturer’s instructions. DNA digestions with restriction enzymes were performed at 37 °C overnight, and complete digestion was confirmed by 1% agarose gel electrophoresis. DNA fragments without the desired removed module were extracted from gels using either the *Monarch® DNA Gel Extraction Kit* (New England Biolabs) or the *QIAquick® Gel Extraction Kit* (QIAGEN, Hilden, Germany). DNA re-ligation reactions were carried out by adding T4 DNA ligase (Promega) and incubating overnight at 10 °C, after which the ligase was heat-inactivated, and the plasmid was transformed into *E. coli* for recovery. The correct removal of sequences after each restriction was verified by both colony PCR and sequencing. For DNA quantifications, after gel electrophoresis, the samples were visualized using the InGenius3 gel documentation system and Genesys software and compared with a standard using *GelAnalyzer* software (www.gelanalyzer.com) (Istvan Lazar Jr., PhD & Istvan Lazar Sr., PhD, CSc).

### *P. pastoris* genetics procedures

Electrocompetent *Pichia pastoris* cells were prepared as described in Invitrogen (2010). The plasmids bearing the desired combination of fusion proteins for expression obtained as described above were linearized using SacI to direct integration of the entire vector in the *P. pastoris* AOX1 promoter. Transformation of yeast was performed with linearized plasmid DNA (>5 µg) by electroporation at 2.5 kV, 25 μF, and 200 Ω. Recovered cells were selected in YPDS plates with 100 μg/mL zeocin and incubated at 28 °C for 5 days. Integration of the correct modular combination plasmid into the *P. pastoris* genome was confirmed through colony PCR using the following primers: 5′ AOX1 (5′- GAC​TGG​TTC​CAA​TTG​ACA​AGC- 3′), 3′AOX1 (5′- GCA​AAT​GGC​ATT​CTG​ACA​TCC- 3′), Plastic-rev (5′- CAC​CAC​TGT​CTG​AAA​TCC​CA- 3′), NB-rev (5′- GTC​CGC​ATA​GTA​TGT​GTA​GC- 3′), HRP-rev (5′- TTG​TCG​TAG​AAG​GTA​GGC​GT- 3′), and *AviTag-rev (*5′- GAT​GTC​GTT​TAG​GCC​ACT​AG- 3′).

### κNB and κNB–HRP induction and purification

For each κNB expression, a single colony was inoculated in a starter culture of 20 mL BMGY and grown at 28 °C with 250 rpm agitation. Cells were then centrifuged, resuspended to OD = 1 in BMMY, and incubated at 28 °C with agitation at 250 rpm for 72 h, adding 1% methanol every 24 h. After 72 h, the culture was centrifuged at 3000 *g* for 10 min, and the supernatant was frozen until purification. κNBs were purified from 200 mL of the culture supernatant using a 1 mL Ni-NTA affinity column, as described by Idrovo-Hidalgo et al. (2024). Fractions that contained protein eluted with 300 mM imidazole were identified using the Bradford assay and run on a 15% SDS-PAGE (κNB) or 10% SDS-PAGE (κNB–HRP). Fractions were pooled, and purified protein was dialyzed against TBS (20 mM Tris-HCl and 150 mM NaCl, pH 7.4). Pure protein was quantified by UV spectroscopy, measuring absorbance at 280 nm using the following extinction coefficients: for κNB = 32,890.00 M^-1^ cm^-1^ and κNB–HRP = 45,840.00 M^-1^ cm^-1^, assuming fully reduced cysteines. The κNB size was verified by SDS-PAGE runs (15% for κNB and 10% for κNB–HRP) stained with Coomassie Brilliant blue.

### Western blots

To verify NBs, expression gels were transferred to PDVF membranes for Western blotting during 80 min at 100 V in 20% methanol, 25 mM Tris, and 192 mM glycine buffer. Western blot membranes were blocked with 3% low-fat milk and incubated with a mouse anti-His (1:7500, Roche, Basel, Switzerland) as the primary antibody and a goat anti-mouse-HRP (1:15,000, Sigma) as the secondary antibody; alternatively, mouse anti-GFP (1:1000, Roche) was used when testing the κNB–HRP as the secondary antibody. Membranes were revealed using the *SuperSignal® West Pico Chemiluminescent Substrate* (Thermo Fisher Scientific) and visualized in a GeneGnome imaging system using GenSys software (10 min exposure with 1 min intervals or 20 min exposure with 2 min intervals).

### κNB deglycosylations

High-mannose glycans were removed from purified κNBs under native conditions using 14.4 mU of EndoH, produced in *P. pastoris* in-house, as described by Idrovo-Hidalgo et al. (2024). For glycan removal under denaturing conditions, κNBs were first denatured by heating at 95 °C for 10 min in denaturing buffer (0.5% SDS and 40 mM DTT) and then incubated with 1.44 mU of EndoH at 37 °C for 1 h. Analysis was performed through either SDS-PAGE or Western blot.

### Size exclusion–high-performance liquid chromatography

Purified κNBs were injected into a Superose-6 column (GE Healthcare, Chicago, Illinois, United States) coupled to a JASCO HPLC using a UV–VIS UV-2075 detector. The running buffer composition was 20 mM Tris-HCl, 100 mM NaCl, and 1 mM EDTA, pH 7.0. The experiment was run for 70 min at room temperature (∼25 °C), with a flow set to 0.4 mL/min, and elution was monitored at 280 nm.

### ELISA analysis

Flat-bottom 96-well ELISA plates (Greiner Bio-One, catalogue: 675061, Kremsmünster, Austria) were coated with human, llama, or mouse blood serum diluted in carbonate buffer pH 9.6 (0.1 M carbonate buffer pH 9.6: 16 mL of solution A [0.2 M Na_2_CO_3_], 34 mL of solution B [0.2 M NaHCO_3_] (16.8 g per liter), and 50 mL H_2_O) and incubated overnight at 4 °C. After adsorption, plates were washed three times with water and once with PBS-T (0.14 M NaCl, 2.7 mM KCl, 10 mM Na_2_HPO_4_·7H_2_O, 1.4 mM K_2_HPO_4_, and 0.1% Tween 20; pH 7.4). Blocking was performed with 3% milk in PBS-T for 1 h at room temperature. All subsequent incubations were carried out under the same conditions and followed by identical washing steps. NBs were diluted to the desired concentrations in 1.5% low-fat dry milk in PBS-T and added to the wells. To evaluate κNB specificity for mouse serum, 1:100 and 1:1,000 dilutions were used, and to assess its detection limit, a 1:6,000 dilution was tested, followed in both cases by incubation using a commercial HRP-conjugated anti-His tag antibody (HRP Anti-6X His tag^®^ antibody, Abcam, catalogue: ab1187, Cambridge, United Kingdom) at a 1:5000 dilution. When testing the κNB–HRP recognition of mouse serum in a direct assay, mouse serum (1:2400 dilution) was adsorbed to a plate, and κNB–HRP was applied at concentrations ranging from 0.57 to 36.4 nM. When testing κNB–HRP as a secondary antibody using an indirect assay, plates were coated with 4 μg/mL of SARS-CoV-2 RBD (Arbe et al., 2020; Idrovo-Hidalgo et al., 2024). Anti-RBD mouse serum (1:300 dilution) was used as the primary antibody, and κNB–HRP was applied at concentrations ranging from 0.57 to 36.4 nM. After the final incubation and washing, the HRP substrate TMB (TMB Substrate Reagent Set, BD Biosciences, catalogue BDB-555214, Franklin Lakes, New Jersey, United States) was added. Absorbance was measured at 450 nm using an Infinite M Plex plate reader (Tecan, Männedorf, Switzerland).

### Dot blot

For the dot blot assay, 0–10 µg of purified RBD protein (Arbe et al., 2020; Idrovo-Hidalgo et al., 2024) was spotted onto a PVDF membrane and allowed to dry completely at room temperature. Membrane blocking, washing, and detection were performed as described for Western blotting. Anti-RBD mouse serum (1:1000) was used as the primary antibody, and κNB-HRP (1:3000) was used as the secondary antibody.

### HRP fast activity test

To assess the activity of the HRP enzyme expressed in *P. pastoris* expressing κNB–HRP, 10 μL of 1/100 dilutions of supernatants from the induced culture medium supernatant were mixed with 5 μL of the TMB substrate. A positive result was rapidly visualized by a color change from clear to blue (progressing to brown upon saturation).

### Statistical analysis and computational work

ANOVA with two factors and replication was performed in Microsoft Excel, using an α value of 0.05 (5%) . Detection limit or end-point titer was defined as the NB concentration that produced a signal at 450 nm that was greater than the cut-off value, which was set at three times the average blank value. The antibody EC_50_ value was calculated using GraphPad Prism 5 software. Nonlinear regression (curve fit) and sigmoidal dose-response (variable slope) models were selected.

## Data Availability

The original contributions presented in the study are included in the article/Supplementary Material; further inquiries can be directed to the corresponding author.
