# Supplementary material for: Production of high-affinity glycosylated anti-mouse conjugated nanobodies in Pichia pastoris
Source: Front Bioeng Biotechnol. 2025 Oct 15;13:1673481. doi: 10.3389/fbioe.2025.1673481 (PMC12569646; doi:10.3389/fbioe.2025.1673481)
Supplement: Supplementary file 1 [file DataSheet1.pdf]

## Supplementary material

# Production of high-affinity glycosylated anti-mouse conjugated nanobodies in *Pichia pastoris*


**Sofía Orioli[1], Javier Santos[1,2,3], Lorena I. Ibañez[3,4], Cecilia D'Alessio[1,3]***

[1] Universidad de Buenos Aires, Facultad de Ciencias Exactas y Naturales, Departamento de Fisiología y Biología Molecular y Celular, Instituto de Biociencias, Biotecnología y Biología Traslacional (iB3), Buenos Aires, Argentina.

[2] Universidad de Buenos Aires, Facultad de Ciencias Exactas y Naturales, Departamento de Química Biológica.

[3] Consejo Nacional de Investigaciones Científicas y Técnicas (CONICET), Buenos Aires, Argentina.

[4] CONICET- Universidad de Buenos Aires. Instituto de Química Física de los Materiales, Medio Ambiente y Energía (INQUIMAE), Buenos Aires, Argentina

***To whom correspondence should be addressed**: Cecilia D'Alessio, Universidad de Buenos Aires, Facultad de Ciencias Exactas y Naturales, Departamento de Fisiología y Biología Molecular y Celular, Instituto de Biociencias, Biotecnología y Biología Traslacional (iB3), Intendente Güiraldes 2160, C1428EGA, Buenos Aires, Argentina. E-mail: cdalessio@fbmc.fcen.uba.ar


## Supplementary Figure S1.

**Plastic binding sequence**

**α secretion factor**

```
MRFPSIFTAVLFAASSALAAPVNTTTEDETAQIPAEAVIGYSDLEGDFDVAVLPFSNSTNNGLLFINTTI
ASIAAKEEGVSLEKREAEAEFGTVHWDFRQWWQPSENLYFQSEFGTQVQLQESGGGWVQPGGSLR
LSCAASGFTFSDTAMMWVRQAPGKGREWVAAIDTGGGYTYYADSVKGRFTISRDNAKNTLYLQM   κNB
NSLKPEDTARYYCAKTYSGNYYSNYTVANYGTTGRGTQVTVSSGTASMQLTPTFYDNSCPNVSNIV
RDIIVNELRSDPRIAASILRLHFHDCFVNGCDASILLDNTTSFRTEKDAFGNANSARGFSVIDRMKAA
VESACPGTVSCADLLTIAAQQSVTLAGGPSWRVPLGRRDSLQAFLDLANANLPAPFFTLPQLKDSFR   HRP
NVGLNRSSDLVALSGGHTFGKSQCRFIMDRLYNFSNTGLPDPTLNTTYLQTLRGLCPLNGNLSALVD
FDLRTPTIFDNKYYVNLEEQKGLIQSDQELFSSPDATDTIPLVRSFANSTQTFFNAFVEAMDRMGNIT
PLTGTQGQIRRNCRVVNSNSDLASGGLPETGHHHHHH**TSGLNDIFEAQKIEWHESGHHHHHH**
```

**His-Tag** **Avi-Tag**

**Figure S1. Complete amino acid sequence encoded by the pPICZαA-Plastic-κNB-HRP-Hisx6-AviTag vector shown in Figure 1D ("Modular plasmid").** Note that α secretion factor is removed during yeast secretion an it is not present in mature proteins. Also, note that Hisx6 tag and Avi-Tag cannot coexist in the same protein. In this work, NBs were expressed without the plastic-binding sequence, fused either to a Hisx6 tag alone (κNB) or to both HRP and Hisx6 (κNB-HRP).

**Supplementary Figure S2**

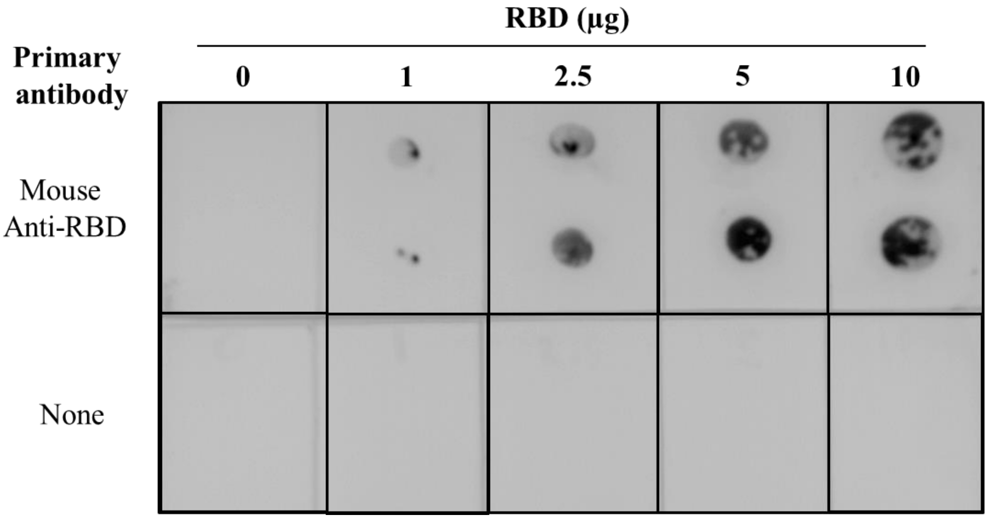


**Figure S2. Use of κNB-HRP as a secondary antibody in a dot blot.** Dot blot assay with 0–10 µg of purified RBD, incubated with mouse anti-RBD primary antibody (1:1000, top) or without primary antibody (bottom), followed by detection with κNB-HRP (1:3000).

*Supplementary Figure S3*

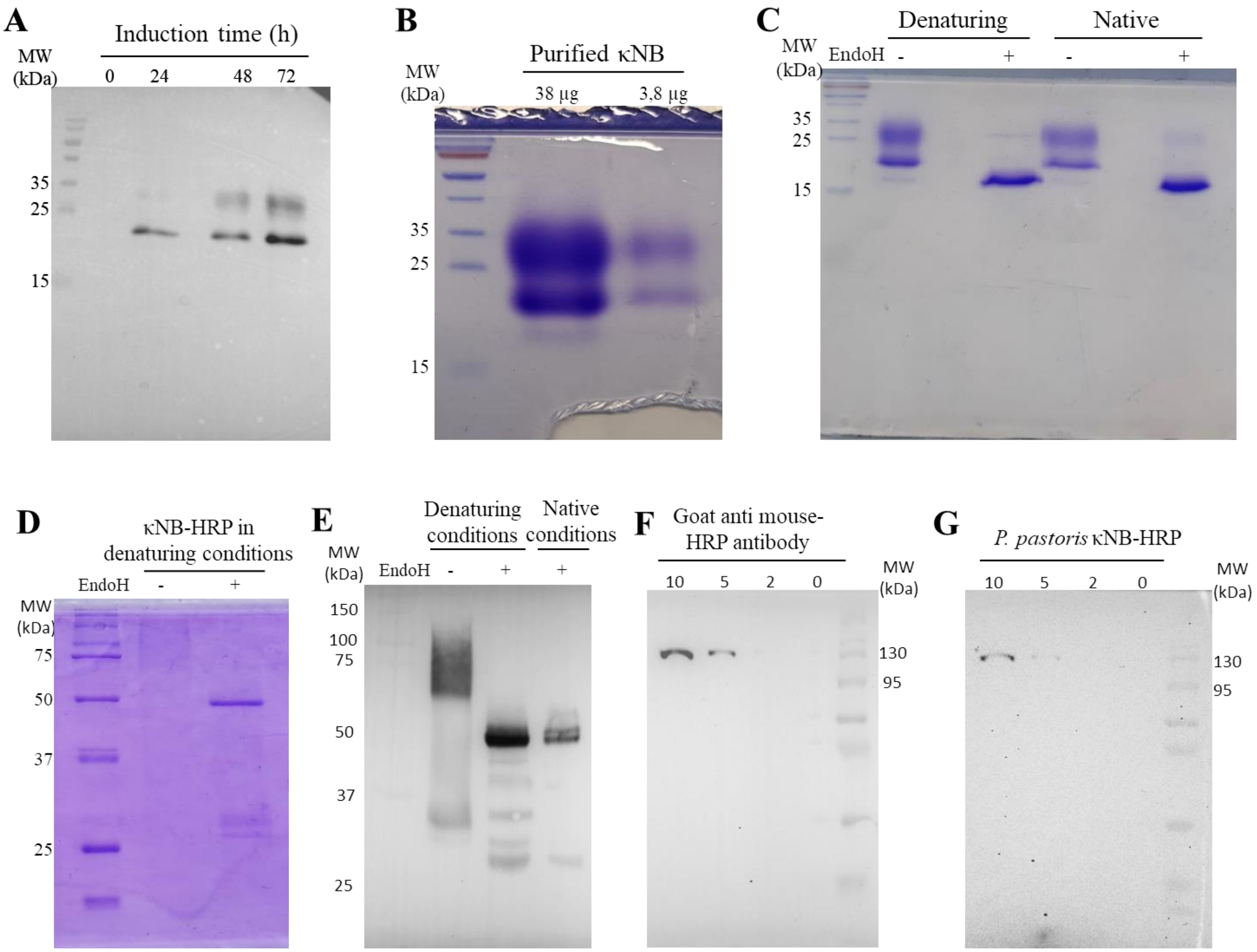


**Figure S3. Full length SDS-PAGE and western blot images corresponding to the main manuscript.** (A) Western blot from Figure 2A. (B) SDS-PAGE from Figure 2B. (C) Corresponds to Figure 3A. (D) to Figure 5A. (E) to Figure 5B. (F) to Figure 7A. (G) to Figure 7B.
